# Can splitting influence corporate strategy? Exploring the effects of TMT faultlines on internationalization speed

**DOI:** 10.1371/journal.pone.0324505

**Published:** 2025-05-28

**Authors:** Jia Lv, Jie Zhou, Yajun Liu

**Affiliations:** 1 Law School, Southwestern University of Finance and Economics, Chengdu, Sichuan, China; 2 School of International Business, Southwestern University of Finance and Economics, Chengdu, Sichuan, China; Duke University, UNITED STATES OF AMERICA

## Abstract

On the basis of upper echelons theory, this paper examines the effect of TMT faultlines on internationalization speed and considers the contextual factors that may affect the focal relationship. We focus on A-share listed manufacturing enterprises in the Shanghai and Shenzhen stock markets from 2010 to 2016 to explore the nexuses. The conclusions indicate that TMT faultlines exerts a significant negative influence on the speed of internationalization. However, the presence of executives shareholding and dynamic capabilities serve to mitigate the negative impact to some extent. These findings hold both theoretical and practical significance for Chinese firms’ internationalization in the context of the ‘double-loop strategy’.

## 1. Introduction

In light of the profound advancement of globalization, an increasing number of Chinese enterprises have commenced the implementation of internationalization strategy. In contrast to the gradual and methodical process of overseas expansion observed in developed countries, the surge in the proportion of OFDI by Chinese enterprises reflects a more accelerated pattern. This highlights the importance of ‘speed’ in the study of international expansion. The recent years have seen Chinese enterprises grappling with a more complex and fierce competition, influenced by factors including geopolitical tensions, intensified trade conflicts and global corona-virus pandemic. Thus, it is of great significance to investigate the factors influencing the pace of internationalization in order to improve quality and enjoy superior performance. However, different from enterprises in developed countries, Chinese companies are confronted with a number of challenges pertaining to relatively outdated management style and scarce resources for internationalization. The core goal for Chinese enterprises is to address these issues and achieve a sustainable and irreplaceable competitive advantage. Under such condition, top management team (TMT) consisting of well-educated and high-quality talents, is a solid foundation and key solution. Therefore, exploring the influence of TMT on internationalization speed provides a significant and practical implication for Chinese enterprises who are in a critical period of transition to high-quality development.

In 1984, Hambrick and Mason initially proposed the upper echelons theory, thereby introducing the executive team perspective to the field of organizational management. Since then, research on corporate executives has shifted from the individual level to the team level [1]. Early studies on the executive team level mainly focused on the impact of team heterogeneity and diversity, but the results yielded inconclusive findings. Some scholars have argued that team heterogeneity not only provides enterprises with rich and diverse resources, but also effectively enhances performance by facilitating in-depth and comprehensive communication among members [2,3]. Nevertheless, some studies found that team heterogeneity may result in conflict and antagonism, which in turn reduce the quality and efficiency in decision-making [4,5]. Therefore, existing research on the influence of team heterogeneity has not reached a unified conclusion. The reason behind this phenomenon is that such studies typically treat different dimensional characteristics of TMT members separately, focusing solely on the effect of differences based on one single characteristic. However, such treatment may potentially lead to two issues. Firstly, it overlooks the reality that individual behavior may be influenced by multiple characteristics simultaneously. Secondly, the combined impact of multiple characteristics is not simply the sum of individual dimension but rather a higher-dimensional aggregation effect that ultimately influences organizational behavior and decision-making. In response to this, Lau and Murnighan [6] extended the concept of team heterogeneity and initially proposed the concept of group faultlines by utilizing a combination of measures instead of relying on a single characteristic to assess team composition in a more comprehensive manner.

Faultline, a virtual dividing line, separates the team into a number of internally homogeneous but mutually heterogeneous subgroups based on one or more characteristics. Given that team members exhibit varying degrees of differences across multiple dimensions, based on the similarity-attraction paradigm, members with common characteristics will instinctively gather together. This clustering forms distinct subgroups that fragment the executive team, thereby creating faultlines. The strength of TMT faultlines depends on two aspects: the internal consistency of subgroups and the differences between subgroups. When members within a subgroup share the same or similar characteristics across multiple dimensions, the internal consistency is enhanced, and thus the aggregation effect of multidimensional characteristics becomes more pronounced. Similarly, when the differences between subgroups are greater, it indicates a higher level of internal division and alienation within the executive team. As a result, the overall intensity of the faultlines will be strengthened. In another words, the executive team can be distinctly and clearly divided into several different subgroups. Since TMT faultline is more inclined towards the internal structure of the executive team, it is able to elucidate the influence of TMT in a more systematic and comprehensive way. Additionally, existing research has also confirmed that TMT faultlines have stronger explanatory power for team and organization-level outcomes [7].

Nowadays, empirical research has demonstrated that TMT faultlines significantly influence corporate development and behavior, including internationalization strategy, innovation performance, green technological innovation, growth performance, strategic change and internal control quality [8–13]. In addition, the impact is observed as positive, negative as well as non-linear. On one hand, social categorization and social identity theory suggests that executive members tend to categorize themselves and others based on perceptions and comparisons of different characteristics. This process gradually forms a sense of ‘inside vs outside’, which may lead to conflicts and disagreements among members, impeding the exchange and sharing of knowledge and information. As a result, TMT faultlines negatively affect corporate performance [14–16]. On the other hand, on the basis of information processing theory, diverse membership characteristics provide a rich pool of knowledge, resources and information for decision-making, as well as facilitating organizations to decide expeditiously. In addition, in-depth exchange and discussion will motivate members to adopt a more objective and prudent attitude which is conducive to corporate performance as well. Beyond that, Thatcher [17] identified a U-shaped relationship between faultlines and team conflict, as well as an inverted U-shaped nexus with team performance. The finding suggests that team performance is optimal when faultline is at a moderate level. Similarly, Liu [18] and Ma [19] also clarified an inverted U-shape relationship between TMT faultlines strength and technological innovation. However, they found that physiological faultline does not significantly affect the level of green technological innovation.

The internationalization process represents a key area of focus in international business research. Early theories paid less attention to the temporal dimension of the internationalization process or generally examined performance and decision-making processes only through a simple static treatment. The concept of internationalization speed was first proposed by Vermeulen and Barkema [20], who classified internationalization into three dimensions: scope, speed and rhythm. Scope refers to the geographical scope of overseas expansion, while speed focuses on the degree of change in internationalization over a specified period. Rhythm, on the other hand, examines the coherence and regularity in a firm’s expansion trajectory. However, there is no consensus on the definition of internationalization speed. At present, there are two principal categories: initial speed and post-entry speed. The former focuses on the period between a firm’s foundation and its initial internationalization [21,22], indicating the point at which the firm begins its foreign expansion. It measures ‘how early’ rather than ‘how fast’ the internationalization process begins. The latter examines the growth of overseas operations following internationalization [23]. Prashantham and Young [24] introduced the concept of post-entry speed and measured it in terms of both financial and operational indicators. Existing literature has examined the factors influencing internationalization speed from four main dimensions: individual and team, organizational, inter-organizational and institutional. At the individual and team level, individual managers and top management teams play a pivotal role in determining strategic decisions and future direction. However, they are bounded rationality and are prone to make totally different decisions based on different characteristics. Consequently, individual’s experience, education background, attitude and risk preference may affect firm’s internationalization speed [25–27]. At the organizational level, internal factors such as firm’s resources and learning capabilities can influence internationalization speed [28,29]. Scarce and redundant organizational resources represent an important source of competitive advantage, which can effectively improve the ability to deal with risk and facilitate firms to accelerate overseas expansion [30]. In addition, firm’s technological and marketing capabilities also play a positive role in influencing internationalization speed [31]. The inter-organizational level examines factors from social network perspective. Empirical studies have demonstrated that firms in different network structures exhibit varying degrees of difficulty in accessing information related to foreign operations. This ultimately affects their propensity to internationalize [27]. Finally, besides internal factors, host country and home country environment may also exert a significant influence on firm’s strategic decisions. This involves factors such as political, economic, legal and cultural elements. Extant literature has primarily investigated the impact of distinct institutions [26], but the conclusions remain inconclusive.

A review of the existing literature on TMT faultlines and internationalization speed reveals a divergence of opinion regarding the influence of TMT faultlines on firm’s strategic choices. In addition, different political, economic and cultural environment can also influence member’s decision-making process. Thus, this paper focuses on Chinese multinational enterprises so as to better understand Chinese specific situation. We also attempt to provide targeted and realistic guidance to enterprises for the formulation and implementation of overseas strategies. Meanwhile, the existing literature of top management team primarily examines the static characteristics of the members. However, this paper extends this field by analyzing the joint effect of multiple characteristics, exploring the influence of internal composition on firm’s international strategy. Furthermore, we also introduce two specific contextual variables, namely executives shareholding and dynamic capabilities, for in-depth discussion.

The structure of this study is organized as follows. Section 2 introduces the theoretical background and major hypotheses. Section 3 describes our data sources and variable measurements. Section 4 shows our empirical results, including baseline regression and all the robustness tests. Section 5 is the discussion and conclusion of the article.

## 2. Theory and hypotheses

### 2.1. Theoretical background

The Upper Echelons Theory initially emerged from in-depth explorations of leadership and decision-making within the fields of organizational behavior and management. Prior to this, Taylor primarily focused on how to enhance efficiency through improvements in organizational structure and processes, viewing decision-making as a process based on complete rationality. With the development of behavioral science, scholars began to pay attention to the behavior and decision-making processes of individuals within organizations, arguing that individual behaviors in organizations can significantly influence organizational strategies. Influenced by this shift, Dearborn [32] proposed the theory of bounded rationality, which focuses on the differences in how individual decision-makers process information, suggesting that individuals are limited by their cognitive abilities when facing complex decisions. Building on this, scholars started to examine the impact of individual factors in the decision-making process and further explored the role and influence of leaders within organizations. This was aimed at revealing the limitations of traditional theories in explaining the influence of top management decisions on organizational behavior.

Hambrick and Mason’s proposal of upper echelons theory opened a new chapter in the study of leadership theories and organizational behavior. This theory is based on the assumption of “bounded rationality”, positing that human decision-making is constrained by limitations in information processing, time, and cognitive capacity, thus preventing fully rational decisions. When confronted with complex and uncertain situations, individuals are unable to access complete “knowledge” of all information and can only make personalized interpretations and judgments based on limited information and cognitive abilities. These personalized interpretations are a reflection and manifestation of team members’ experience, value and personality and existing research has confirmed that members’ background characteristics are effective proxy variables for their cognitive patterns [33]. Thus, given the same business environment and relevant supporting information, executives may make totally different decisions because of divergence in perceptions of risk, environment and interest motivation. Different characteristics of executives in terms of professional background, age, gender, education and tenure are the main reasons for these divergences because they shape executive members’ past experience, values and cognitive perceptions, which ultimately have a significant impact on business strategic decisions [34,35].

In summary, the Upper Echelons Theory follows the research logic of “characteristics-cognition and decision-making,” integrating leaders’ characteristics, cognitive patterns, and strategic behaviors and decisions into one theoretical model. It comprehensively proposes that TMT members, as the main formulators of strategic decisions, possess different background characteristics that fully reflect their unique experiences, values, and cognitive patterns, thereby forming personalized interpretations of organizational contexts and ultimately influencing and altering corporate strategic choices. This theoretical perspective also lays a solid foundation for this paper to deeply explore the impact of faultlines formed by the multidimensional characteristics of TMT members on the corporate strategic decision of internationalization speed.

Driven by the improvement of science and technology, enterprise management gradually requires richer knowledge reserves, higher technological level and larger scale of human resources. Under such condition, traditional division of labor mode alone no longer meets the requirement. As a result, the division of labour has been further refined. Ownership and management of enterprises have been gradually separated. Jensen and Meckling [36] put forward the principal-agent theory in 1976, opening up relevant academic research. Principal-agent theory mainly examines the contractual relationship between professional managers and the owners, with managers providing specialized managerial decision-making services for which firm’s shareholders pay. Ideally, professional managers are motivated by the same goal of maximizing share value and firm profits. However, under realistic situations, there is a serious problem of moral hazard. Executive members may turn to pursue personal interests other than overall interests out of subjective or objective factors. In addition, due to the withdrawal of shareholders from the actual management level, they mainly receive relevant information from professional managers, which in turn causes serious information asymmetry problem as well. Therefore, in order to effectively mitigate such problems, scholars have proposed a variety of targeted remedies, such as increasing the number of agents to achieve effective regulation, establishing a sound reputation mechanism for agents or improving executive shareholding level as a salary incentive. All solutions aim to provide common goals for executives and enhance their work motivation so as to alleviate the principal-agent problem.

Dynamic capability theory is proposed to facilitate enterprises to better adapt to changes in external environment. To some extent, it compensates for the shortcomings of resource base view and core capability view, and has now become an important theoretical lens for analyzing sustainable competitive advantage. Dynamic capability, an ability to identify, acquire, integrate and reconfigure resources in response to external changes, is essential for enterprises’ survival and development. Existing research primarily examines three types of dynamic capability: knowledge [37,38], convention [39] and ability [40,41]. According to the theory, dynamic access to information and resources, accurate formation of innovative capability in response to changes is a key way to improve performance and obtain sustainable competitive advantages for firms [42].

### 2.2. TMT faultlines and internationalization speed

Upper echelons theory posits that top management team is responsible for formulating strategic choices and charting future direction [4]. In contrast to developed countries, emerging economies face greater challenges in entering foreign markets due to costs and latecomer disadvantages. Thus, it is crucial for them to hasten internationalization process to compensate for resource disparity and competitive disadvantages. However, compared to domestic market, overseas markets present a more complex and uncertain business environment because it requires not only a larger scale of resources and information, but also higher administrative ability of executive team. Therefore, executive teams are of great significance to firm’s development. Based on social identity theory and similar-attraction paradigm [43], executive members tend to categorize themselves and others through examining whether they have similar characteristics or not. Consequently, top management team will be divided into several internally homogeneous but mutually heterogeneous subgroups. Subgroup members will commit a strong sense of identity towards their own circles but hold a negative perception towards other groups out of stereotypes, eventually causing serious fragmentation and reduced overall trust [15].

There are three reasons why TMT faultlines hamper internationalization speed. Firstly, TMT faultlines impede the construction of a platform for sharing information and resources within the firm. Members from different professional backgrounds collect diverse information, bringing a wealth of information and resources to the organization [44]. However, fragmentation can result in members being more inclined to share information with their own groups and make strategic decisions based on the interests of subgroups rather than overall team. Thus, there is a natural exclusion and mistrust among members, which in turn results in a failure to communicate and share relevant information between subgroups [45]. In addition, mistrust can also lead to conflict and a lack of internal cohesion, which is detrimental to the achievement of overall business goals. When the resources are limited, each subgroup will band together to compete for more benefits, crowding out and suppressing others that do not share the same interests or goals. Consequently, vicious competition between subgroups, inefficient resource allocation and non-essential internal losses may exert a negative influence on internationalization speed. Secondly, TMT faultlines may cause emotional conflict, which affects the quality and efficiency of communication within the team and reduces members’ sense of psychological security. Based on the social identity theory, the formation of an ‘us-them’ consciousness leads to inefficient and meaningless discussions among members due to stereotypical emotional conflicts. As a result, executive teams are unable to reach agreement on organizational decisions or unwilling to correct mistakes in a timely manner [46]. In a word, the reluctance to collaborate with each other increases the risk of international expansion and negatively affects internationalization speed. Thirdly, TMT faultlines can cause staffing disruptions and exacerbate pay differentiation. As the faultline strength increases, staffing within the executive team becomes extremely difficult because subgroups are formed by personal characteristics which have strong social stability. Thus, it is difficult to fundamentally change internal structure through external management measures. In addition, as different subgroups pursue different goals, with faultline strength increasing, the wage gap between subgroups will gradually widen. Consequently, it will trigger a crisis of trust and ultimately lead to inefficiency in decision-making, which is detrimental to advancing internationalization speed.

Hypothesis 1. TMT faultlines will negatively affect internationalization speed.

### 2.3. The moderating role of executives shareholding

Based on the principal-agent theory, there is a contractual relationship between professional managers and shareholders. Ideally, executive managers provide professional managerial decision-making services for which shareholders pay compensation. However, under realistic situation, there exists moral hazard problems and information asymmetry due to different interests and goals [47]. The value pursued by executive members as employees are different from those of shareholders, in addition, the existence of TMT faultlines can further exacerbate the divergence of interests, which is not conducive to the long-term development of the company. However, existing literature has clarified that executives shareholding, as a long-term salary incentive, can effectively resolve the conflicts between executives and shareholders [36]. The influence can be analyzed through two aspects. Firstly, executives shareholding can enhance members’ sense of identity and weaken the stereotypes and conflicts. Based on the realistic conflict theory, goal congruence is an effective motivation for friendly attitude towards different groups. Only when there exists a same goal, can members from different subgroups establish a cooperative relationship [48]. Therefore, executives shareholding provides members with a clear and well-defined goal, i.e., maximization of enterprise’s share value [49]. And the goal can bridge the faultlines, facilitating a buffer against conflict and confrontation between subgroups. It can also diminish the prejudice and disparity between members, encouraging a harmony environment for knowledge exchange and information sharing. Eventually, it is conducive to team spirit and internationalization process. Secondly, executives shareholding can effectively improve the motivation of team members. Expectancy theory suggests that behavior is influenced by desired outcomes. When the benefits are significant and the probability of success is higher, the incentive effect is stronger. Executives shareholding, as a long-term incentive, can fully satisfy the core demands of members and link their personal interests with the performance of the company. Thus, it can stimulate members’ enthusiasm and encourage them to choose strategies conducive to the long-term enterprise performance. In a word, since executives shareholding provides team members with common goal and strong motivation to bridge fragmentation between subgroups, it can effectively mitigate the negative influence of TMT faultlines on internationalization speed.

Hypothesis 2. Executives shareholding positively moderates the negative effect of TMT faultlines on internationalization speed.

### 2.4. The moderating role of dynamic capability

Teece et al. [42] proposed dynamic capability theory to explain how firms are able to obtain sustainable competitive advantage. Dynamic capability refers to the ability to identify, acquire, integrate and reconfigure knowledge and resources in order to cope with challenges and uncertainties. Existing research has justified that dynamic capabilities can positively affect internationalization process. Firstly, the coordination and integration ability inherent in dynamic capabilities can effectively enhance enterprises’ resource integration and strategic management ability [41]. With dynamic capabilities, firms are able to acquire diverse resources and design suitable organizational structure for overseas production and operation, and thus reducing the cost of internationalization. Secondly, enterprises with high dynamic capabilities are able to identify key information, explore new knowledge and develop new markets in an uncertain environment. It is realized by building a large customer network worldwide and developing extensive information access channels. Concurrently, enterprises are able to monitor changes in the external environment timely, guiding firms to make specific changes in order to meet customer needs and increase international sales. Finally, firms are able to obtain sustainable competitive advantages due to adapting ability [50]. Enterprises with high dynamic capabilities tend to have flexible organizational structures that is conducive to adaption. It enables firms to change promptly according to specific situation and develop new products that align with the needs of international markets. As a result, multinationals can maintain core competitiveness and facilitate a steady advancement of overseas expansion.

In the context of executive teams, dynamic capabilities are able to activate TMT faultlines exerting a ‘positive’ effect. Bezrukova [51] has found that faultlines may have positive effect through facilitating sharing of resources, as well as implementing of multi-perspective analysis. Both behaviors are conducive to superior performance. Similarly, Mello and Ruckes [52] also justified that teams with diverse attributes are able to access more information and resources compared to homogeneous groups. Firms with high dynamic capabilities tend to invest more in R&D and innovation. Under such organizational culture that encourages innovation, executive team members are more inclined to engage in open dialogue, addressing issues of information asymmetry and decision-making inefficiency. Consequently, it can attenuate the negative influence of TMT faultlines on internationalization speed.

Hypothesis 3. Dynamic capability positively moderates the negative effect of TMT faultlines on internationalization speed.

## 3. Research design

### 3.1. Sample and data sources

This paper selects A-share listed manufacturing multinationals in Shanghai and Shenzhen stock markets from 2010–2016 to investigate the influence of TMT faultlines on internationalization speed, and tests the moderating effect of executives shareholding and dynamic capabilities. We choose A-share manufacturing enterprises for three reasons. Firstly, upper echelons theory suggests that studies of executive attributes based on the same industry may reach the least biased results [4]. Secondly, manufacturing industry accounts for a large proportion of Chinese multinational investment, serving as the basis of modern industrial system. Thus, choosing manufacturing industry offers an appropriate empirical setting for investigating the influence of TMT faultlines. Thirdly, based on the data availability, selecting enterprises listed in A-share market enables us to obtain adequate information about the company through multiple channels.

The information about manufacturing multinationals and the location of overseas subsidiaries is primarily derived from the Wind database. The data on internationalization speed is sourced from China Stock Market Accounting Research (CSMAR) and Wind database while data about executives characteristics mainly comes from the CSMAR and CNRDS database. In addition, we collect information about executives shareholding and dynamic capabilities from the Wind and iFinD databases. All other control variables information are manually collected from the CSMAR database. The final sample obtains 2846 firm-year observations by excluding the enterprises that are ST, *ST and PT in the current year, as well as those with missing indicators or incomplete data.

### 3.2. Definitions and measurements of main variables

#### Internationalization speed.

Speed refers to how rapidly a firm expands into an international market during a certain period. Following the measurement proposed by Shi and Prescott [53] and Chen [54], this paper uses speed to denote a five-year average growth ratio of the amounts of international revenue for a focal firm. We first regressed the natural logarithm of a focal firm’s international revenue and an index variable of the time period in years, with time serving as an independent variable. Then, the antilog of the regression coefficient ($b_{2}$), capturing the growth rate of the firm’s international revenue, is used as the measurement of its speed of international expansion. The basic growth equation for this measure is given by:


$$\;\ln\left({OI}_{t}\right)=b_{1}+b_{2}t+\delta$$ (3-1)

where ${OI}_{t}$ is a firm’s international revenue in year t; δ is residual; and the antilog of $b_{2}$ is the internationalization speed.

#### TMT faultlines.

Following Thatcher, Jehn and Zanutto [17], we measure the faultline strength, ${Fau}_{g}$, as how a group splits into subgroups. In the case of teams divided according to a dichotomous model, faultline strength is quantified by dividing the inter-group sum of squares between the subgroups by the sum of squares of the team as a whole. ${Fau}_{g}$ takes values between 0 and 1, with a maximum value of 1 when the two subgroups are perfectly homogeneous internally. The basic equation for this measure is given by:


$$\begin{array}{c} \;{Fau}_{g}=\frac{\sum_{j=1}^{q}\sum_{k=1}^{2}n_{k}^{g}{\left({\overset{―}{x}}_{jk}-{\overset{―}{x}}_{j}\right)}^{2}}{\sum_{j=1}^{q}\sum_{k=1}^{2}\sum_{i=1}^{n_{k}^{g}}{\left(x_{ijk}-{\overset{―}{x}}_{j}\right)}^{2}}\;,\;g=1,\;2,\;\ldots ,\;s\; \end{array}$$ (3-2)

where $X_{ijk}$ represents the value of the $j^{th}$ characteristic of the $i^{th}$ member of subgroup k, $\overset{―}{X_{j}}$ is the overall group mean of characteristic j, $\overset{―}{X_{jk}}$ is the mean of characteristic j in subgroup k, $n_{k}^{g}$ is the number of members of the $k^{th}$ subgroup (k = 1,2) under split g. We consider only group splits in which the size of each subgroup k has at minimum two members. Our measure consists of four attributes of executive members. The categorical indicators of gender, educational background, functional background and age are classified as follows: gender (male = 1, female = 2); educational level (high school or below=1, college = 2, university = 3, master’s degree = 4, PhD = 5) [55]; functional background (production roles = 1, service roles = 2, support roles = 3); age (no more than 30 years old = 1, 31–40 years old = 2, 41–50 years old = 3, more than 51 years old = 4).

#### Moderators.

Following Chang’s [56] study, this paper measures executives shareholding as ratio of the number of shares held by executive members to total share capital of the company. For dynamic capability, extant researches have proposed several different measures, but no single measure methodology is superior to all others [57]. For example, Cohen and Levinthal [58] utilizes R&D intensity to measure while Zahra and George [22] believe that dynamic capability is related to knowledge creation and utilization, and thus using absorptive capacity to measure the construct. This paper adopts the second method and utilizes the number of patents granted by the firm in the current year as a proxy variable for absorptive capacity to measure dynamic capability.

#### Control variables.

This paper controls for several factors that may affect internationalization speed from the executive team level, firm level and industry level respectively. (1) TMT size: measured as the total number of executive members in the year. (2) Diversification: calculated by Herfindel index, suggesting the share of the $i^{th}$ industry in total sales. The higher the index, the lower the degree of diversification. (3) Firm size: operationalized as the log of total assets at the end of the year. (4) Firm growth: measured by the growth rate of the operating income. (5) Asset liability ratio: measured as the ratio of total liabilities to total assets. (6) Dual: set as a binary variable to measure this construct. If CEO is also the chairman of the board of directors, the value is 1, otherwise 0. (7) Ownership: set as a dummy variable to clarify the type of enterprise. State-owned enterprise takes the value 1, otherwise 0. Finally, we control for the year effect by generating year dummy variables.

### 3.3 Econometrical model

This paper empirically tests the above hypotheses through three theoretical models. In order to test the influence of TMT faultlines on internationalization speed, we design the following model for analysis.


$$\;{SPEED}_{it}=\alpha_{0}+\alpha_{1}{TMTFau}_{it}+\alpha_{2}Z_{it}+\lambda_{t}+\mu_{it}$$ (3-3)

In addition, we add interaction terms of executives shareholding and dynamic capability with TMT faultlines respectively so as to explore the moderating role of these factors. The regression model is shown as follows.


$${SPEED}_{it}=\beta_{0}+\beta_{1}{TMTFau}_{it}+\beta_{2}{MShare}_{it}+\beta_{3}{TMTFau}_{it}\times {MShare}_{it}+\beta_{4}Z_{it}+\lambda_{t}+\mu_{it}$$ (3-4)

$${SPEED}_{it}=\theta_{0}+\theta_{1}{TMTFau}_{it}+\theta_{2}{DC}_{it}+\theta_{3}{TMTFau}_{it}\times {DC}_{it}+\theta_{4}Z_{it}+\lambda_{t}+\mu_{it}$$ (3-5)

where i,t refers to the focal firm and year. ${SPEED}_{it}$ refers to internationalization speed, ${TMTFau}_{it}$ is the TMT faultline strength, ${MShare}_{it}$ represents executives shareholding and ${DC}_{it}\;$refers to firm’s dynamic capabilities. $Z_{it}\;$includes all control variables and $\lambda_{t}$ represents year fixed effect.

## 4. Results

### 4.1. Descriptive analysis

Table 1 presents the results of descriptive statistics and correlation coefficients for the key variables in this paper. As can be seen from Table 1, the mean value of internationalization speed is 1.149. It demonstrates that the overall speed of international expansion in China’s manufacturing industry is relatively fast. However, there is a large gap between the maximum and minimum values, indicating that there is a significant difference among different firms. In terms of the TMT faultlines, over 50% of the enterprises exhibited a strength greater than 0.4, suggesting a prevalence of faultline phenomenon in Chinese listed enterprises. Similarly, there is a notable disparity between different enterprises considering the gap between maximum and minimum values. The correlation coefficient results reveal that all key variables in this paper exhibit a correlation coefficient less than 0.7. Furthermore, we also conduct a variance inflation factor (VIF) test in order to mitigate the impact of multicollinearity on the results. The average VIF value is found to be 1.48, which is considerably lower than the critical value of 10 commonly recognized in international business, further clarifying that multicollinearity is not a serious issue in this study.

**Table 1:** Descriptive statistics and correlation.

Variables	1	2	3	4	5	6
1. International speed	1.000					
2. TMT faultlines	-0.134	1.000				
3. Dynamic capacity	0.182	-0.515	1.000			
4. Executives shareholding	0.122	0.016	0.006	1.000		
5. TMT size	-0.163	-0.102	-0.034	-0.019	1.000	
6. Diversification	0.017	0.130	-0.054	0.033	-0.009	1.000
7. Firm size	-0.320	-0.017	-0.118	-0.055	0.271	-0.119
8. Growth	-0.004	0.012	-0.008	0.006	0.003	0.006
9. Asset liability ratio	-0.126	-0.055	-0.002	0.030	0.123	-0.115
10. CEO duality	0.008	-0.013	0.018	-0.055	-0.020	-0.019
11. Ownership	-0.077	-0.080	0.053	0.013	0.031	-0.062
Mean	1.149	0.420	2.033	5.082	6.496	0.806
SD	0.161	0.155	0.641	4.817	2.129	0.261
Min	0.415	0.068	1.006	0.001	4.000	0.030
Max	3.032	0.999	8.268	67.146	23.000	1.000
	7	8	9	10	11	
7. Firm size	1.000					
8. Growth	0.034	1.000				
9. Asset liability ratio	0.473	0.030	1.000			
10. CEO duality	-0.198	-0.012	-0.153	1.000		
11. Ownership	0.299	-0.014	0.308	-0.166	1.000	
Mean	21.887	0.252	0.388	0.330	0.361	
SD	1.120	2.813	0.201	0.470	0.480	
Min	17.641	-0.791	0.008	0.000	0.000	
Max	26.465	140.241	2.861	1.000	1.000	

### 4.2. Baseline results

Table 2 presents the influence of TMT faultlines on internationalization speed. Model 1 solely tests the control variables, whereas Model 2 incorporates TMT faultlines to assess the focal relationship in this paper. The results show that the coefficient of the TMT faultlines is significantly negative at 1% level (β = -0.156, p = 0.000), indicating that an increase in the faultline strength will lead to sharp conflicts between team members, which in turn exerts a negative impact on the internationalization speed of enterprises. Thus, hypothesis 1 is supported.

**Table 2:** Baseline results.

	Model 1	Model 2
TMT faultlines		-0.156^***^ (0.000)
TMT size	-0.006^***^ (0.000)	-0.007^***^ (0.000)
Diversification	-0.012 (0.298)	0.001 (0.990)
Firm size	-0.047^***^ (0.000)	-0.046^***^ (0.000)
Growth	0.0003 (0.763)	0.0004 (0.701)
Asset liability ratio	0.024 (0.152)	0.018 (0.268)
CEO duality	-0.019^***^ (0.003)	-0.020^***^ (0.002)
Ownership	-0.002 (0.768)	-0.002 (0.732)
Observations	2846	2846
Fixed effect	Yes	Yes

Note: The coefficients in the table are multiple linear regression coefficients; P values are in parentheses;

*** p < 0.01,

** p < 0.05,

* p < 0.1

Table 3 shows the results of testing the moderating effects of executives shareholding and dynamic capabilities. Model 1 and 3 incorporate the moderators, executives shareholding and dynamic capabilities respectively. In addition, model 2 and 4 add the interaction terms of TMT faultlines and two moderators in order to explore the moderating effect on the focal nexus. According to model 2, it can be found that the moderating effect of executives shareholding is significant and positive at 1% level (β = 0.011, p = 0.001), thereby corroborating hypothesis 2. Meanwhile, the results of model 4 demonstrates that dynamic capabilities exert a significantly positive influence on the negative relationship between TMT faultlines and internationalization speed (β = 0.107, p = 0.001). It suggests that if enterprises possess higher level of dynamic capabilities, they can better cope with the negative influence of TMT faultlines that the nexus will be significantly attenuated. Thus, hypothesis 3 is completely supported.

**Table 3:** Moderating effect of executives shareholding and dynamic capability.

	Model 1	Model 2	Model 3	Model 4
TMT faultlines	-0.158^***^ (0.000)	-0.215^***^ (0.000)	-0.107^***^ (0.000)	-0.288^***^ (0.000)
Executives shareholding	0.003^***^ (0.000)	-0.001 (0.481)		
TMT faultlines^*^Executives shareholding		0.011^***^ (0.001)		
Dynamic capability			0.023^***^ (0.000)	-0.008 (0.467)
TMT faultlines^*^Dynamic capability				0.107^***^ (0.001)
TMT size	-0.0071^***^ (0.000)	-0.0073^***^ (0.000)	-0.007^***^ (0.000)	-0.007^***^ (0.000)
Diversification	-0.002 (0.883)	-0.001 (0.933)	-0.0001 (0.999)	-0.003 (0.770)
Firm size	-0.044^***^ (0.000)	-0.043^***^ (0.000)	-0.044^***^ (0.000)	-0.044^***^ (0.000)
Growth	0.0003 (0.729)	0.0003 (0.736)	0.0004 (0.711)	-0.0003 (0.750)
Asset liability ratio	0.013 (0.442)	0.009 (0.567)	-0.016 (0.325)	0.017 (0.305)
CEO duality	-0.017^***^ (0.005)	-0.018^***^ (0.004)	-0.019^***^ (0.002)	-0.019^**^ (0.002)
Ownership	-0.003 (0.665)	-0.002 (0.699)	-0.004 (0.574)	-0.004 (0.552)
Observations	2846	2846	2846	2846
Fixed effect	Yes	Yes	Yes	Yes

Note: The coefficients in the table are multiple linear regression coefficients; P values are in parentheses;

*** p < 0.01,

** p < 0.05,

* p < 0.1

**Fig 1:**
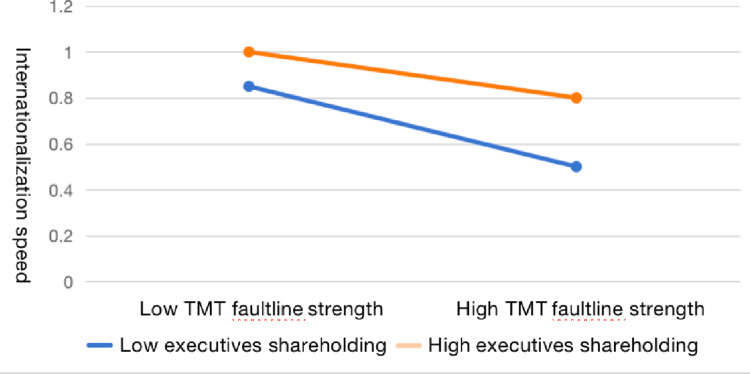
Interaction plot of executives shareholding.

**Fig 2:**
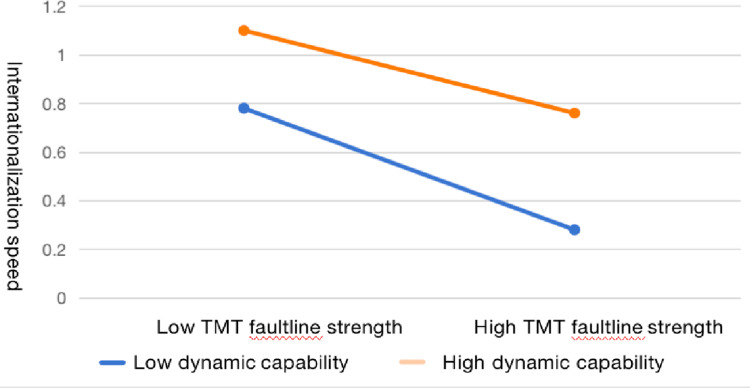
Interaction plot of dynamic capabilities.

In addition, this paper also draws a plot of the interactions (Fig 1 and 2) in order to accurately observe the effect of the moderating influence. We add or subtract the standard deviation from the sample mean to form the high and low TMT faultlines samples and conduct the regressions respectively. As illustrated in Fig 1, the slope of high executives shareholding is flatter than that of low executives shareholding, suggesting that executives shareholding as a salary incentive can significantly mitigate the negative effect of TMT faultlines. Similarly, Fig 2 illustrates that high dynamic capabilities can notably attenuate the focal nexus compared to the low level of dynamic capabilities.

### 4.3 Robustness check

#### Robustness check by changing measurements of independent and dependent variables.

Besides the approach proposed by Thatcher [17], another commonly used approach is the FLS algorithm proposed by Shaw [59]. This approach takes both inter-group variability and intra-group consistency into consideration, and utilizes the aggregated indicator to measure faultline strength. The calculation formula is presented as follows:


      FLS=IA ×(1−CGAI) (4-1)

where IA refers to the degree of consistency of other attributes within the subgroup when the team is divided according to a certain characteristic. Furthermore, (1-CGAI) indicates the degree of variability between subgroups in terms of other individual attributes. In total, FLS takes values from 0 to 1, with a closer value to 1 suggesting a greater strength of faultline.

In terms of internationalization speed, in addition to the previous measurement proposed by Shi and Prescott [53], another reliable measurement is to assess the change of the degree of internationalization over a one-year period [31]. And the degree of internationalization is operated as the ratio of overseas sales revenue to the firm’s total sales revenue. Thus, this study changes the measurement of independent and dependent variables to conduct a robustness check. The corresponding results are demonstrated in Table 4, in which Model 1 shows the result of changing TMT faultlines measure and Model 2 presents that of changing internationalization speed. It is evident that the results in Table 4 are consistent with the baseline regression, further justifying that the results of this paper are robust and reliable.

**Table 4:** Robustness check by changing measurements of key variables.

	Model 1	Model 2
TMT faultlines	-0.164^***^ (0.000)	-0.296^***^ (0.000)
TMT size	-0.007^***^ (0.000)	-0.0002 (0.071)
Diversification	0.0003 (0.973)	-0.003^***^ (0.000)
Firm size	-0.046^***^ (0.000)	0.0001 (0.659)
Growth	0.0004 (0.979)	-0.0001 (0.641)
Asset liability ratio	0.018 (0.273)	0.001 (0.688)
CEO duality	-0.021^***^ (0.001)	0.001 (0.347)
Ownership	-0.002 (0.741)	-0.0004^***^ (0.481)
Observations	2846	2846
Fixed effect	Yes	Yes

Note: The coefficients in the table are multiple linear regression coefficients; P values are in parentheses;

*** p < 0.01,

** p < 0.05,

* p < 0.1

#### Robustness check by changing measurements of moderators.

In the baseline regression, we use the number of patents granted by the enterprise in the current year as a proxy variable for dynamic capabilities. To further test the robustness of the findings, this paper draws on Cohen and Levinthal’s [58] study and utilizes R&D intensity to measure dynamic capabilities. R&D investment intensity is defined as the percentage of annual R&D expenditures in the firm’s operating revenues. The results are reported in Table 5, in which Model 1 incorporates the moderators, dynamic capability, while Model 2 adds the interaction terms of TMT faultlines and internationalization speed. Since the results remain consistent, indicating that the findings of this paper are robust and reliable.

**Table 5:** Robustness check by changing measurements of dynamic capabilities.

	Model 1	Model 2
TMT faultlines	-0.134^***^ (0.000)	-0.190^***^ (0.000)
Dynamic capability	0.111^***^ (0.006)	-0.126 (0.200)
TMT faultlines^*^Dynamic capability		0.711^***^ (0.008)
TMT size	-0.0071^***^ (0.000)	-0.0073^***^ (0.000)
Diversification	0.0002 (0.988)	-0.003 (0.803)
Firm size	-0.045^***^ (0.000)	-0.045^***^ (0.000)
Growth	0.0004 (0.687)	0.0003 (0.690)
Asset liability ratio	0.017 (0.293)	-0.019 (0.257)
CEO duality	-0.019^***^ (0.002)	-0.019^***^ (0.002)
Ownership	-0.002 (0.699)	-0.003 (0.685)
Observations	2846	2846
Fixed effect	Yes	Yes

Note: The coefficients in the table are multiple linear regression coefficients; P values are in parentheses;

*** p < 0.01,

** p < 0.05,

* p < 0.1

### 4.4. Heterogeneity analysis

Under the background of Chinese socialist market economy, state-owned enterprises (SOEs) play a pivotal role in economic development, while non-state-owned enterprises (Non-SOEs), due to their large numbers, also exert a significant influence on economic growth. Since state-owned enterprises are mainly controlled by the state, they are in a position to obtain relevant resources and policy support easier. Furthermore, they have a stronger risk resilience to better survive and develop in international markets. However, compared with non-state-owned enterprises, they also bear greater social responsibility and are more susceptible to the policy change. Therefore, different ownership modes may bring different pursuit of interests to executive members. This paper thus re-tests the focal relationship and moderating effects by taking different ownership structures into consideration. Table 6 presents the results of the grouping heterogeneity test. As shown in Table 6, the effects of different firm properties on the focal relationship and the moderating effect are comparable, with only minor differences in the coefficients. In addition, the results of the heterogeneity analysis align with those of the full sample, reinforcing the stability and reliability of the findings presented in this paper.

**Table 6:** Heterogeneity analysis by differential ownership structure.

	State-owned enterprises			Non-State-owned enterprises		
	Model 1	Model 2	Model 3	Model 4	Model 5	Model 6
TMT faultlines	-0.134^***^ (0.000)	-0.219^***^ (0.000)	-0.334^***^ (0.002)	-0.167^***^ (0.000)	-0.228^***^ (0.000)	-0.257^***^ (0.001)
Executives shareholding		-0.001 (0.562)			-0.002 (0.413)	
TMT faultlines ^*^Executives shareholding		0.018^***^ (0.009)			0.012^***^ (0.006)	
Dynamic capability			-0.013 (0.471)			-0.005 (0.731)
TMT faultlines ^*^Dynamic capability			0.155^***^ (0.006)			0.076^*^ (0.078)
TMT size	-0.0072^***^ (0.001)	-0.007^***^ (0.001)	-0.0071^***^ (0.001)	-0.0073^***^ (0.000)	-0.008^***^ (0.000)	-0.0074^***^ (0.000)
Diversification	0.014 (0.437)	0.009 (0.614)	0.008 (0.650)	-0.008 (0.565)	-0.008 (0.574)	-0.010 (0.471)
Firm size	-0.048^***^ (0.000)	-0.046^***^ (0.000)	-0.043^***^ (0.000)	-0.043^***^ (0.000)	-0.041^***^ (0.000)	-0.043^***^ (0.000)
Growth	-0.0052 (0.358)	-0.0051 (0.362)	-0.0049 (0.385)	0.0004 (0.632)	0.0004 (0.668)	0.0004 (0.664)
Asset liability ratio	0.063^**^ (0.021)	0.063^**^ (0.021)	-0.061^**^ (0.027)	-0.010 (0.633)	-0.23 (0.276)	-0.010 (0.616)
CEO duality	0.005 (0.670)	0.012 (0.309)	0.007 (0.555)	-0.029^***^ (0.000)	-0.028^***^ (0.000)	-0.029^***^ (0.000)
Observations	1026	1026	1026	1820	1820	1820
Fixed effect	Yes	Yes	Yes	Yes	Yes	Yes

Note: The coefficients in the table are multiple linear regression coefficients; P values are in parentheses;

*** p < 0.01,

** p < 0.05,

* p < 0.1

## 5. Discussion

Based on the upper echelons theory, principal-agent theory and dynamic capability theory, this paper empirically examines the influence of TMT faultlines on internationalization speed using data from A-share listed manufacturing enterprises from 2010–2016. We also explore the mitigating effect of executives shareholding and dynamic capabilities. The results show that TMT faultlines can negatively affect internationalization speed. The greater the faultlines strength, the stronger the fragmentation between different subgroups. It will significantly intensify the conflicts between members and eventually lead to the inability to build a platform for information exchanging and knowledge sharing within the organization. Consequently, the quality and efficiency of organizational can be seriously affected, and thus impeding the pace of corporate internationalization. Nevertheless, there are solutions for such problem. According to our results, executives shareholding and dynamic capabilities can effectively mitigate the negative influence of faultlines. This is because both factors are able to provide a common goal for different subgroups and promote the improvement of corporate resource integration ability and decision-making efficiency, thus contributing to accelerated internationalization speed.

Compared with prior studies, this paper has three aspects of theoretical contributions as following. First of all, existing literature on executive characteristics has primarily focused on their impact on corporate static performance and strategic decision-making, with relatively few studies focusing on the dynamic process of internationalization. However, the internationalization path of Chinese enterprises is characterized by a leapfrog process, which highlights the significance of the “speed” of internationalization. In addition, in terms of internationalization speed, Chinese scholars have mainly concentrated on the relationship between internationalization strategy and corporate performance, while neglected the exploration of the antecedents of internationalization strategy. In order to fill this theoretical void, this paper thus introduces TMT faultlines as an explanatory variable to examine their impact on dynamic corporate performance—internationalization speed in detail. Additionally, unlike previous heterogeneity and diversity studies, this paper introduces “faultline” construct to examine the combined effect of multiple characteristics of TMT members. It not only enriches the research on executive characteristics and team composition, but also represents a further application and extension of the Upper Echelons Theory. Secondly, based on the principal-agent theory, this paper also investigates the moderating effect of executive shareholding. By considering the influence of corporate power structure and equity incentives on the relationship between managerial cognition and strategic decision-making, this paper further extends the Upper Echelons Theory and provides additional evidence on organizational behavior and strategic choice. Finally, existing research on dynamic capabilities has not yet reached a unified conclusion, and most studies have treated it as an explanatory variable or a dependent variable, with relatively few studies considering it as a moderating variable. This study explores the moderating effect of dynamic capability on corporate performance and the “activation” impact on TMT faultlines, further providing a new perspective for understanding the role of dynamic capabilities in corporate internationalization.

The findings of this paper also suggest three practical implications for enterprises. Firstly, enterprises should pay greater attention to the phenomenon of TMT faultlines that the joint effect of members characteristics may exert an influence on internationalization speed. Top management team is the primary decision-maker in the organization, determining the future direction of the long-term development. Therefore, it is crucial of enterprises to attach great importance to the process of personnel selection and assignment so as to minimize the likelihood of subgroups formation. Concurrently, enterprises should cultivate a harmonious and open environment for communication, encouraging members to freely express their opinions, knowledge and information. Gradually, this will facilitate a highly cohesive corporate culture and enhance employee’s sense of belonging, significantly mitigating the negative impact of the faultlines. Secondly, it is important to consider the positive influence of executives shareholding as an effective salary incentive. On the one hand, it can provide a common goal for different subgroups, alleviating stereotypes and conflicts among them. On the other hand, it can also enhance executive members’ motivation, and thus improving working efficiency. Thirdly, it is of paramount importance to cultivate the dynamic capability continuously because it enables enterprises to adapt to the complex environment. The higher the dynamic capability, the more the enterprise can rely on its ability to identify, acquire and integrate knowledge resources to advance internationalization process steadily.

This paper examines the drivers and contextual factors affecting internationalization speed. However, there are still some shortcomings. Firstly, the data used in this paper only covers the period from 2010 to 2016, which is a limited time span. In addition, the sample size is relatively small and the industry is limited to the manufacturing industry. Future research could examine the impact of TMT faultlines in other industries by updating the time period and expanding the research sample. Secondly, there are limitations in the measurement of TMT faultlines. This paper only examines four characteristics but did not examine deeper psychological attributes such as race, tenure or cultural background. It may not be able to comprehensively measure the faultlines construct. Future research could further explore deeper attributes in order to comprehensively understand the phenomenon of faultlines. Thirdly, there are limitations in examining the internationalization process of firms. Based on existing research, overseas expansion encompasses three dimensions: internationalization speed, rhythm and scope [20]. This paper, however, only examines the single dimension of speed. Therefore, future research could explore the effects of TMT faultlines on the rhythm and scope dimensions in order to achieve a comprehensive consideration.

## Supporting information

S1 Raw Data All raw data used in this paper. (XLSX)
